# Novel Antiplatelet Activity of Minocycline Involves Inhibition of MLK3-p38 Mitogen Activated Protein Kinase Axis

**DOI:** 10.1371/journal.pone.0157115

**Published:** 2016-06-06

**Authors:** Joseph W. Jackson, Meera V. Singh, Vir B. Singh, Letitia D. Jones, Gregory A. Davidson, Sara Ture, Craig N. Morrell, Giovanni Schifitto, Sanjay B. Maggirwar

**Affiliations:** 1 Department of Microbiology and Immunology, University of Rochester Medical Center, Rochester, New York, United States of America; 2 Aab Cardiovascular Research Institute, University of Rochester Medical Center, Rochester, New York, United States of America; 3 Department of Neurology, University of Rochester Medical Center, Rochester, New York, United States of America; University of Missouri-Kansas City, UNITED STATES

## Abstract

Platelets play an essential role in hemostasis and wound healing by facilitating thrombus formation at sites of injury. Platelets also mediate inflammation and contain several pro-inflammatory molecules including cytokines and chemokines that mediate leukocyte recruitment and activation. Not surprisingly, platelet dysfunction is known to contribute to several inflammatory disorders. Antiplatelet therapies, such as aspirin, adenosine diphosphate (ADP) antagonists, glycoprotein IIb/IIIa (GPIIb/IIIa) inhibitors, and anticoagulants such as warfarin, dampen platelet activity at the risk of unwarranted bleeding. Thus, the development of drugs that reduce platelet-mediated inflammation without interfering with thrombus formation is of importance to combat platelet-associated disorders. We have shown here for the first time that the tetracycline antibiotic, minocycline, administered to HIV-infected individuals reduces plasma levels of soluble CD40L and platelet factor 4 levels, host molecules predominately released by platelets. Minocycline reduced the activation of isolated platelets in the presence of the potent platelet activator, thrombin, as measured by ELISA and flow cytometry. Platelet degranulation was reduced upon exposure to minocycline as shown by mepacrine retention and flow cytometry. However, minocycline had no effect on spreading, aggregation, GPIIb/IIIa activation, or *in vivo* thrombus formation. Lastly, immunoblot analysis suggests that the antiplatelet activity of minocycline is likely mediated by inhibition of mixed lineage kinase 3 (MLK3)-p38 MAPK signaling axis and loss of p38 activity. Our findings provide a better understanding of platelet biology and a novel repurposing of an established antibiotic, minocycline, to specifically reduce platelet granule release without affecting thrombosis, which may yield insights in generating novel, specific antiplatelet therapies.

## Introduction

Platelets are small, anucleate cells originating from bone marrow-derived megakaryocytes that have been studied classically for their role in thrombosis and wound healing, and, more recently, inflammation [1]. Upon activation, platelets undergo an extensive shape change and promote the release of granule stores and homotypic aggregation [2]. More recent literature has suggested that platelets play a major role in inflammation via release of a variety of pro-inflammatory molecules from platelet granules that contribute to the recruitment and subsequent activation of leukocytes [3]. Platelet dysfunction can therefore lead to the establishment of an inflammatory phenotype in leukocytes including monocyte-platelet aggregates, and endothelial cell activation [4]. Therefore, it is not surprising that platelets have been implicated in a variety of disorders where inflammation plays an important role, such as atherosclerosis and ischemia/reperfusion [5–7], sepsis [8], arthritis [9], diabetes mellitus [10], cerebral malaria [11], Alzheimer’s disease [12], cancer [13] as well as dengue virus [14], hepatitis B virus [15], and Human Immunodeficiency Virus type-1 (HIV) infection [16].

A large amount of work has been performed in finding the “magic bullet” antiplatelet therapy that dampens the harmful effects of platelet activation, but does not result in severe bleeding and hemostatic dysfunction [17]. Presently, several drugs that dampen platelet activity are used clinically. These include aspirin, which blocks cyclooxygenase and thromboxane production, clopidogrel which blocks adenosine diphosphate (ADP) receptor signaling, as well as abciximab and eptifibatide, which block glycoprotein IIb/IIIa (GPIIb/IIIa) integrin signaling and subsequent thrombus formation [2, 18]. Other drugs, such as warfarin, dampen the coagulation cascade, which ultimately inhibits platelet activity and is used clinically for the treatment of thromboembolisms [19]. However, a common drawback for these drugs is the increased risk of bleeding that can occur with long-term use. While this effect may be suitable for the prevention of thrombus formation, a novel approach that selectively dampens platelet-mediated inflammation without resulting in hemostatic dysfunction is needed. In the present work, we have characterized the selective, novel antiplatelet activity of an antibiotic, minocycline.

Minocycline is a tetracycline derivative that has been tested experimentally for its potential use in a variety of disorders, such as rheumatoid arthritis, ischemia/stroke, atherosclerosis, inflammatory bowel disease, and HIV infection, due to its broad-spectrum antibiotic and anti-inflammatory properties [20]. For example, it was found that minocycline treatment may be beneficial for rescuing blood flow in experimental ischaemia-stroke models [21, 22]. Minocycline is also highly lipid soluble and is able to cross the blood-brain barrier (BBB) [23, 24], and thus has been studied extensively as an adjunctive therapy to combat several neurodegenerative disorders including Alzheimer’s disease [25, 26], Huntington’s disease [27], multiple sclerosis [28, 29], and HIV associated neurocognitive disorders [30]. Since minocycline has been shown to inhibit p38 MAPK signaling in some studies [31, 32], and considering that p38 MAPK signaling is involved in platelet activation and degranulation [1, 33–35], we hypothesized that minocycline would exert antiplatelet activity through abrogation of p38 MAPK kinase. The newly characterized antiplatelet activity of minocycline in conjunction with its broad antibiotic and anti-inflammatory properties could serve as an attractive drug for a variety of disorders.

Here we show for the first time that minocycline treatment reduces platelet activation in HIV-infected individuals and in isolated, washed human platelets treated with thrombin *in vitro*. Minocycline exposure reduced platelet degranulation but did not affect platelet spreading, homotypic aggregation, GPIIb/IIIa expression, or thrombus formation. Furthermore, we demonstrate that mixed lineage kinase 3 (MLK3), an upstream MAPKKK involved in p38 signaling, is involved in platelet activation, and that minocycline attenuated the MLK3-p38 MAPK signaling axis, which may provide a mechanism of minocycline’s selective antiplatelet activity. With its broad-spectrum capabilities, minocycline can serve as a newly characterized, selective antiplatelet agent for controlling a wide range of platelet-mediated inflammatory disorders, with no affect on the formation of thrombi and pose little to no risk for unwarranted bleeding defects. Furthermore, this work may yield insights into the generation of novel antiplatelet compounds that specifically block platelet inflammation without affecting hemostasis.

## Materials and Methods

### Ethics Statement

The Research Subjects Review Board at the University of Rochester Medical Center approved studies involving human samples. All study participants were adults and blood samples were obtained after written informed consent, in accordance with the Declaration of Helsinki.

All experiments involving mice were carried out in accordance with the Animal Welfare Act and the National Institute of Health (NIH) guidelines, and the University Committee on Animal Resources of the University of Rochester Medical Center approved this protocol. The facilities of the Vivarium and Division of Laboratory Animal Medicine of the School of Medicine and Dentistry are fully accredited by the Association for the Assessment and Accreditation of Laboratory Animal Care International. For all animal experiments, mice were anesthetized via an intraperitoneal injection of 100 mg/kg ketamine and 10 mg/kg xylazine. All mice were euthanized via cardiac exsanguination and cervical dislocation, and all efforts were made to minimize suffering. Wildtype C57BL/6 mice were purchased from The Jackson Laboratory, Bar Harbor, ME, USA. MLK3 knockout (MLK3 KO) mice were back-crossed for 10 generations to C57BL/6 mice and managed as a homozygous strain [36].

### Patient Samples

Cryopreserved plasma samples from persons (n = 12) with HIV infection were collected at various time points following the indicated treatments: 0 weeks (baseline) and 2 weeks after receiving minocycline (200 mg/day). All patients were receiving combined antiretroviral therapy (cART) at the time of the blood draw as previously described [37]. Whole blood from male and female seronegative patients was also collected in acid citrate dextrose buffered vacutainers (BD Biosciences, San Jose, CA, USA) for subsequent platelet isolation as described previously [38].

### Reagents

Minocycline, thrombin, mepacrine (quinacrine dihydrochloride), thrombin receptor activator peptide 6 (TRAP 6), valproic acid (VPA), and tyrode’s salt solution were all purchased from Sigma Aldrich (Saint Louis, MO, USA). MLK3 inhibitor, CEP1347, was received from Cephalon (West Chester, PA, USA). Phalloidin AlexaFluor 488 was purchased from Life Technologies (Grand Island, NY, USA). HIV Transactivator of transcription (Tat; 101 amino acids in length) was produced by the University of Rochester Center for AIDS Research Basic Biology Core (University of Rochester Medical Center, Rochester NY, USA).

### Aggregation Assay

Platelet aggregation was quantified using a Whole Blood Aggregometer (Chrono-Log, Havertown, PA, USA). Whole blood was collected from consenting human donors in sodium citrate buffered vacutainers (BD Biosciences, San Jose, CA, USA) and centrifuged at 100xg for 10 minutes to obtain platelet rich plasma (PRP). Subsequently, 2 mL of PRP was centrifuged at 1200xg for 10 minutes to obtain platelet poor plasma (PPP) for normalization. PRP was pre-treated with 1 nM, 5 nM, or 10 nM minocycline for 10 minutes and aggregation was initiated with the addition of 5 μM TRAP 6. Data is shown as total aggregation 6 minutes post exposure to TRAP 6.

### ELISA

Platelet factor 4 (PF4) and soluble CD40L (sCD40L) levels were measured from plasma samples collected from HIV-infected individuals, or from supernatants from isolated human platelets from seronegative donors using a human PF4 ELISA kit and sCD40L ELISA kit (both from R&D Systems, Minneapolis, MN, USA) as described previously [39]. PF4 concentrations and sCD40L concentrations are presented as average (±SEM) of the indicated replicates of each sample in pg/mL.

### FeCl_3_ Induced Thrombus Formation

Whole blood was collected from twelve week old wildtype C57BL/6 control donor mice via cardiac exsanguination. Platelets were then isolated and fluorescently labeled with 10 μM calcein green-AM (Life Technologies, Grand Island, NY, USA) for a total time of 30 minutes. The cells were either treated with control (PBS; n = 6) or treated with 10 nM minocycline (n = 6) for 5 minutes prior to being retro-orbitally injected into the respective four to five week old WT “recipient” mice. Alternatively, four to five week old WT mice were given either two injections of sterile filtered 1X PBS or minocycline (100 mg/kg) (~ 12 hr apart) prior to retro-orbital injection of a fluorescent platelet specific antibody, GPIb-DyLight488 (Cat# X488, Emfret Analytics, Eibelstadt, Germany), to assess the affect of minocycline on thrombosis *in vivo*. The mesenteric artery was exposed and injury was induced by topical application of a 3 mm^2^ piece of Whatmann paper wetted with 15% FeCl_3_ for 45 seconds. Thrombus formation of fluorescently labeled platelets was recorded for 20 minutes or until total thrombotic occlusion and visualized with a 20x objective on a 37°C stage warmer via a Nikon Eclipse TI fluorescent microscope (Nikon Instruments Inc., Melville, NY, USA).

### Flow Cytometry

Platelet activation was assessed by expression of the indicated markers. Briefly, 100 μl of platelet resuspension was either fixed with 4% paraformaldehyde and subsequently stained with 3 μl anti-CD61 AF647 (AbD Serotec, UK) and 10 μl anti-CD62P FITC (P selectin; BD Biosciences, San Jose, CA, USA), or stained directly (no fixative) with 10 μl anti-Pac-1 FITC (BD Biosciences, San Jose, CA, USA) and subsequently acquired using a flow cytometer (Accuri C6, Accuri Cytometers, MI, USA). Platelets were initially gated on CD61 expression and then subsequently analyzed for expression of CD62P or Pac-1.

For CD62P analysis from mouse experiments, nine to eleven week old wildtype C57BL/6 or MLK3 knockout (MLK3 KO) mice were treated (or left untreated) with 100 mg/kg minocycline and allowed to rest for 12 hrs. Both groups of mice were then injected with either saline (n = 4 for each group) or HIV Tat (100 ng/g body weight, n = 4 for each group) retro-orbitally for 1 hr. Siliconized instruments (via Sigmacote; Sigma Aldrich, Saint Louis, MO, USA) were used when handling Tat to avoid loss of the protein. 100 ul of mouse whole blood was then collected and immediately fixed with 4% paraformaldehyde and washed. Subsequently, red blood cells were lysed using ACK Lysing Buffer (Life Technologies, Grand Island, NY, USA), and the remaining cells were stained with 2.5ul CD61 PE and 1ul CD62P FITC (both from BD Biosciences, San Jose, CA, USA) and analyzed as above.

Platelet degranulation was measured by treating platelets with 10 μM mepacrine, followed by the indicated treatments and flow cytometric analysis. Briefly, platelet resuspension was incubated with 10 μM mepacrine for 1 hr prior to activation and minocycline treatment. The platelets were then fixed with 4% paraformaldehyde and stained with anti CD61 AF647 (as mentioned above) and analyzed using an Accuri C6 flow cytometer, or Amis ImageStream (Amnis, Seattle, WA, USA).

### Immunoblot Analysis

After the indicated treatments, whole cell lysates were prepared in ELB buffer (50 mM HEPES (pH 7), 250 mM NaCl, 0.1% Nonidet P-40, 5 mM EDTA, 10 mM NaF, 0.1 mM Na_3_VO_4_, 50 μM ZnCl_2_, supplemented with 0.1 mM PMSF, 1 mM DTT, and a mixture of protease and phosphatase inhibitors). After removal of cellular debris via high-speed centrifugation, lysates were fractionalized on 7.5% SDS PAGE gels and protein was electrophoretically transferred to nitrocellulose membranes (GE Healthcare Bio-Sciences Corporation, Piscataway, NJ, USA). The membranes were then analyzed for immunoreactivity against the following antibodies: rabbit anti human total p38, mouse anti human phosphorylated p38, and phosphorylated activating transcription factor2 (ATF-2) (Cat# 9212s, Cat# 9216s, Cat# 9221; all from Cell Signaling Technology, Danvers, MA, USA). Bound antibodies were detected using species-specific infrared dye (IR-dye) conjugated secondary antibodies and subsequently developed using the LICOR Odyssey Infrared Imager (LICOR Biosciences, Lincoln, NE, USA).

### p38 Kinase Assay

p38 activity was measured in whole cell platelet lysates using a p38 kinase activity kit as per the manufacturer’s instructions (Cell Signaling Technology, Danvers, MA, USA). Briefly, phosphorylated p38 was immunoprecipitated from whole cell lysate using a phosphorylated-p38 MAP kinase monoclonal antibody conjugated to sepharose beads. The beads were then incubated with the substrate, ATF-2, and activity was measured as ATF-2 phosphorylation via immunoblot analysis. Equal protein input was verified by immunoblot analysis of total p38 from the lysate that was used for immunoprecipitation.

### Platelet Spreading

Purified human platelets (1x10^7^ platelets/sample) treated with various concentrations of minocycline for 30 minutes at 37°C were incubated on glass coverslips coated with fibrinogen (150 μg/mL; Sigma Aldrich, Saint Louis, MO, USA) for 45 minutes and blocked with 0.5 μg/mL BSA (Sigma Aldrich, Saint Louis, MO, USA). Platelets were subsequently fixed with 4% paraformeldahyde and washed with PBS. The platelets were stained with phalloidin-Alexa Fluor 488 at a 1:200 dilution in PBS with 0.01% Triton for 45 minutes at room temperature. The coverslips were then mounted on slides and platelet spreading was imaged with a 20x objective using a Ziess Axiovert 200 fluorescent microscope (Ziess, Thornwood, NY, USA) coupled with a Digital CCD Camera (Hamamatsu Photonics, Hamamatsu City, Japan). Images were processed using ImagePro Software (Media Cybernetics, Rockville, MD, USA) and spreading was assessed and quantified based on of platelet morphology and categorized as: 1) fully spread (indicative of lamellopodia), 2) partially spread (indicative of filopodia, but not lamellopodia), or 3) not spread (retaining its discoid shape).

### Scanning Electron Microscopy

Platelets were isolated and spread on fibrinogen coated glass coverslips as outlined above. The coverslips were subsequently fixed with 2.5% glutaraldehyde in a 0.1M sodium cacodylate buffer at 4°C overnight. The cells/coverslips were then post-fixed in 1.0% osmium tetroxide buffer and subsequently transitioned through a graded series of ethanol to 100% and allowed to dry overnight in a fume hood. The coverslips were mounted onto aluminum stubs and sputter coated with gold and imaging was performed using a Ziess Auriga field emission scanning electron microscope coupled to a Gatan digital camera system.

### Statistical Analysis

Statistical analysis was performed using a student’s t test, one way and two way ANOVA followed by Bonferroni’s test for multiple comparisons, or Pearson’s correlation. Data is represented as mean ± SEM, where p<0.05 is considered significant.

## Results

### Minocycline reduces platelet activation in HIV-infected individuals

Minocycline has been widely investigated as a potential therapeutic agent for HIV infection due to its anti-inflammatory properties. A study by Follstaedt et. al. has shown that minocycline reduces the activity of two inflammatory markers, p38 and JNK in the brains of SIV-infected macaques [32]. In addition, it is known that p38 MAPK plays a role in platelet activation and degranulation [1, 35, 40], and that there is excessive platelet activation in HIV-infected individuals [41]. Although a recent study demonstrated minocycline did not improve cognitive function in HIV-infected patients with cognitive impairment [42], we believe that minocycline held promise as an antiplatelet agent.

**Fig 1:**
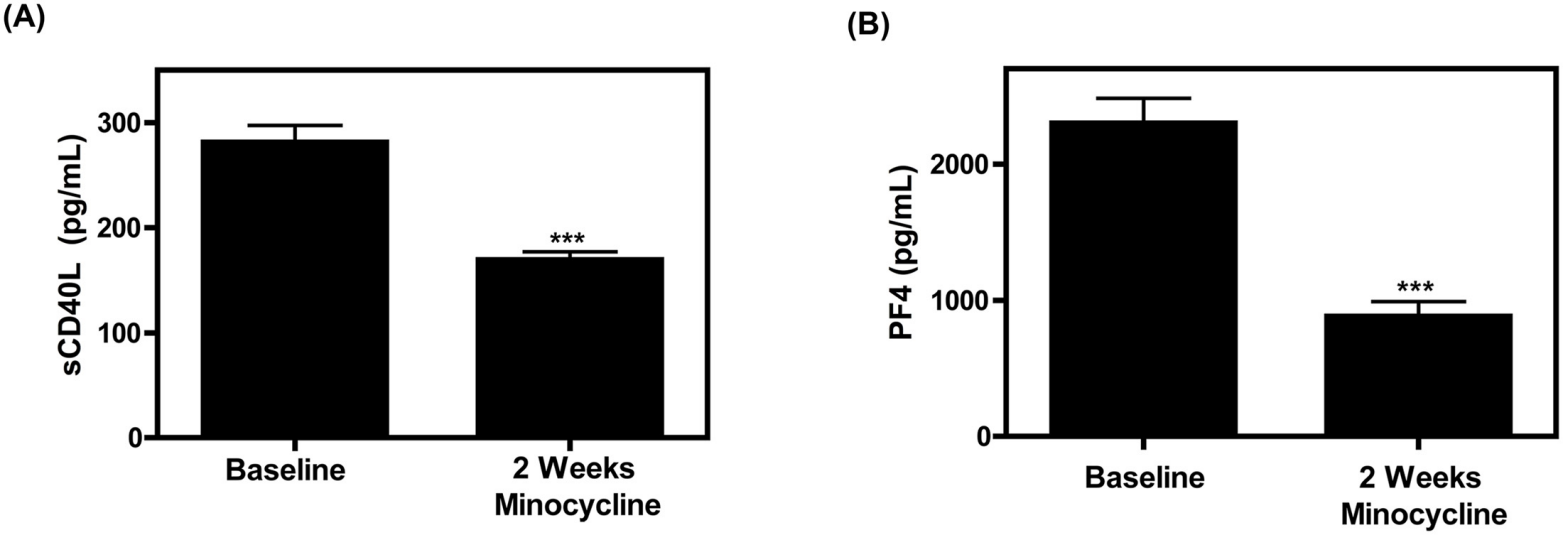
Minocycline treatment reduces sCD40L and PF4 levels in HIV-infected individuals. (A) Plasma sCD40L and (B) plasma PF4 levels were analyzed in HIV-infected individuals (n = 12) receiving cART and an additional treatment of minocycline at a dose of 200mg/day. Baseline corresponds to plasma levels measured at week zero as compared to plasma levels measured after 2 weeks of adjunctive minocycline treatment. The samples were compared using a student’s t test with a 95% confidence interval, where *** denotes p<0.0001.

We therefore investigated whether minocycline treatment inhibited platelet activity. Plasma samples from HIV-infected patients on cART that also received 2 weeks of minocycline therapy at 200mg/day were obtained and platelet activation was assessed via measurement of PF4 and sCD40L levels. The chemokine, PF4, is released from the alpha granules of activated platelets [2], and it is estimated that platelets release approximately 95% of the sCD40L pool [43]. Thus measuring CD40L and PF4 levels in plasma is a means of measuring platelet activation. As shown in Fig 1A and 1B, two weeks of minocycline treatment significantly reduced both sCD40L and PF4 levels in HIV-infected patients as compared to baseline levels (prior to the addition of minocycline treatment), suggesting that minocycline possesses potential antiplatelet activity *in vivo*.

### Minocycline reduces platelet activation in vitro

**Fig 2:**
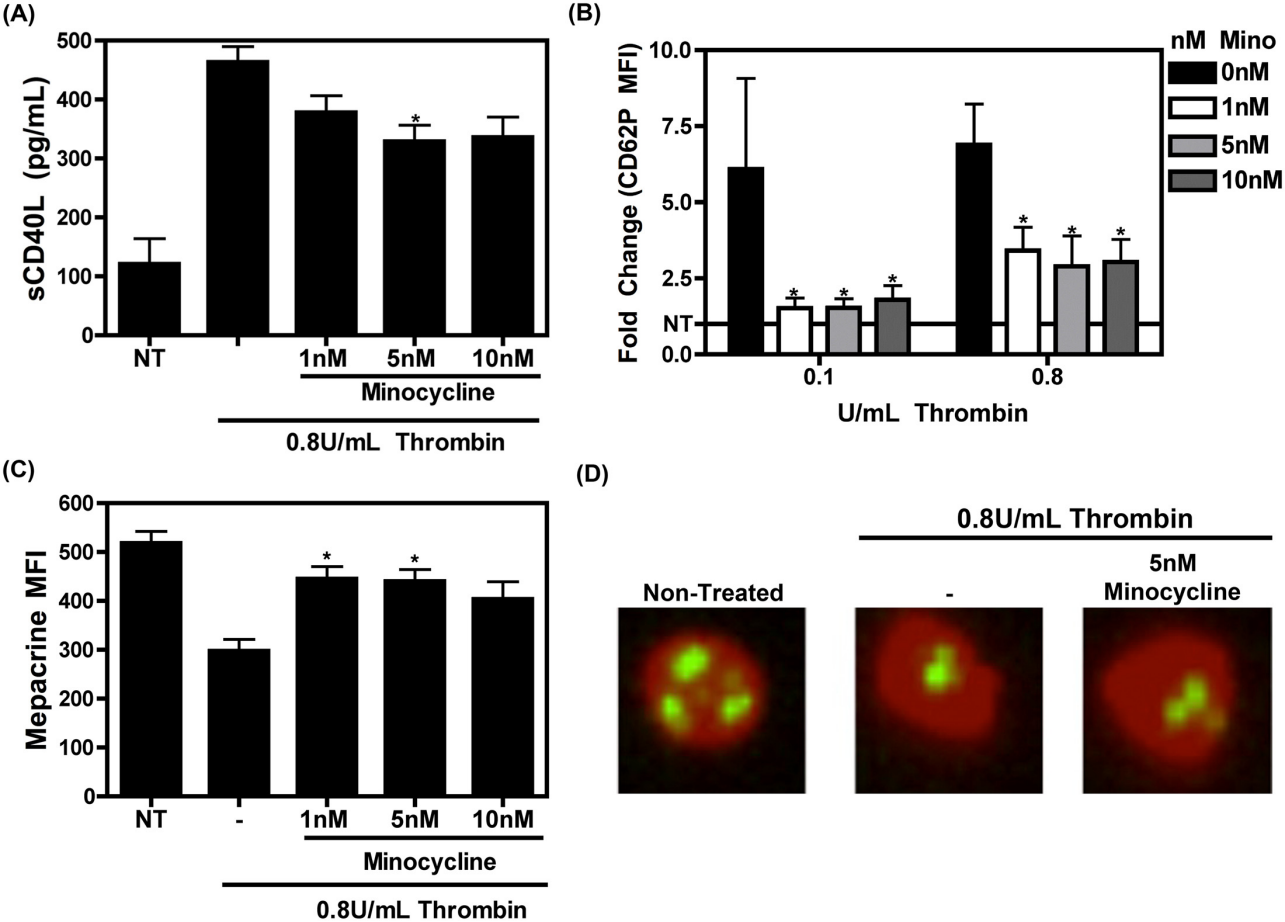
Minocycline reduces activation of human platelets *in vitro*. Platelets from healthy, seronegative donors were isolated and activation was assessed via (A) measurement of sCD40L levels by ELISA after treatment with thrombin alone or together with minocycline, or (B) measurement of CD62P (P selectin; a platelet activation marker) median fluorescent intensity (MFI) by flow cytometry. Data from (B) is presented as fold change of CD62P MFI normalized to non-treated (NT) samples (visualized by horizontal black line set at 1). Data sets for (A) were compared using a one way ANOVA statistical test and data sets for (B) were compared using a two way ANOVA statistical test followed by Bonferroni’s test for multiple comparisons, where * denotes p<0.05. Data shown is representative of three separate experiments. (C) Platelet degranulation was assessed via measurement of mepacrine retention by flow cytometry. The data is representative of three separate experiments and was compared using a one way ANOVA statistical test followed by Bonferroni’s test for multiple comparisons, where * denotes p<0.05. (D) Representative images of mepacrine retention in platelet granules as analyzed by Amnis Image Stream following the indicated treatments. CD61 marks the platelet surface (in red), and mepacrine is stored in platelet dense granules (in green).

Since minocycline treatment reduces sCD40L and PF4 levels in HIV patients, we wanted to validate this phenomenon *in vitro*. Platelets were isolated from HIV negative donors and were treated with thrombin for 1 hr with or without various concentrations of minocycline. Platelet activation was examined by measuring sCD40L levels, which were reduced in minocycline-treated platelets as compared to platelets treated with thrombin alone, again suggesting that minocycline dampens platelet activation (Fig 2A). Although minocycline-mediated sCD40L reduction in washed human platelets is not as striking as that measured from plasma of HIV-infected persons (Fig 1), this may be due to the differences in the experimental setup; comparing sCD40L from human plasma after 2 weeks minocycline therapy as compared to sCD40L levels from isolated human platelets after 1hr minocycline treatment.

Platelet activation can further be assessed via measurement of CD62P (P-Selectin), which is transferred from within alpha granules to the cell surface upon activation. We demonstrate that minocycline pre-treatment reduced CD62P median fluorescent intensity (MFI, presented as fold change) in contrast to platelets treated with different doses of thrombin alone (Fig 2B), reiterating the notion that minocycline reduces thrombin-induced platelet degranulation.

Platelet activation is characterized by a release of both alpha and dense granule contents. Platelet degranulation was therefore further measured by retention of the naturally fluorescent molecule, mepacrine, which is taken up into the dense granules of platelets [44], via flow cytometry and ImageStream. Fig 2C and 2D show that mepacrine is retained in minocycline pre-treated platelets, as compared to mepacrine loss induced by thrombin-only treated platelets. These results suggest that minocycline reduces thrombin-mediated platelet activation and reduces alpha and dense granule content release.

### Minocycline does not reduce platelet spreading and aggregation

**Fig 3:**
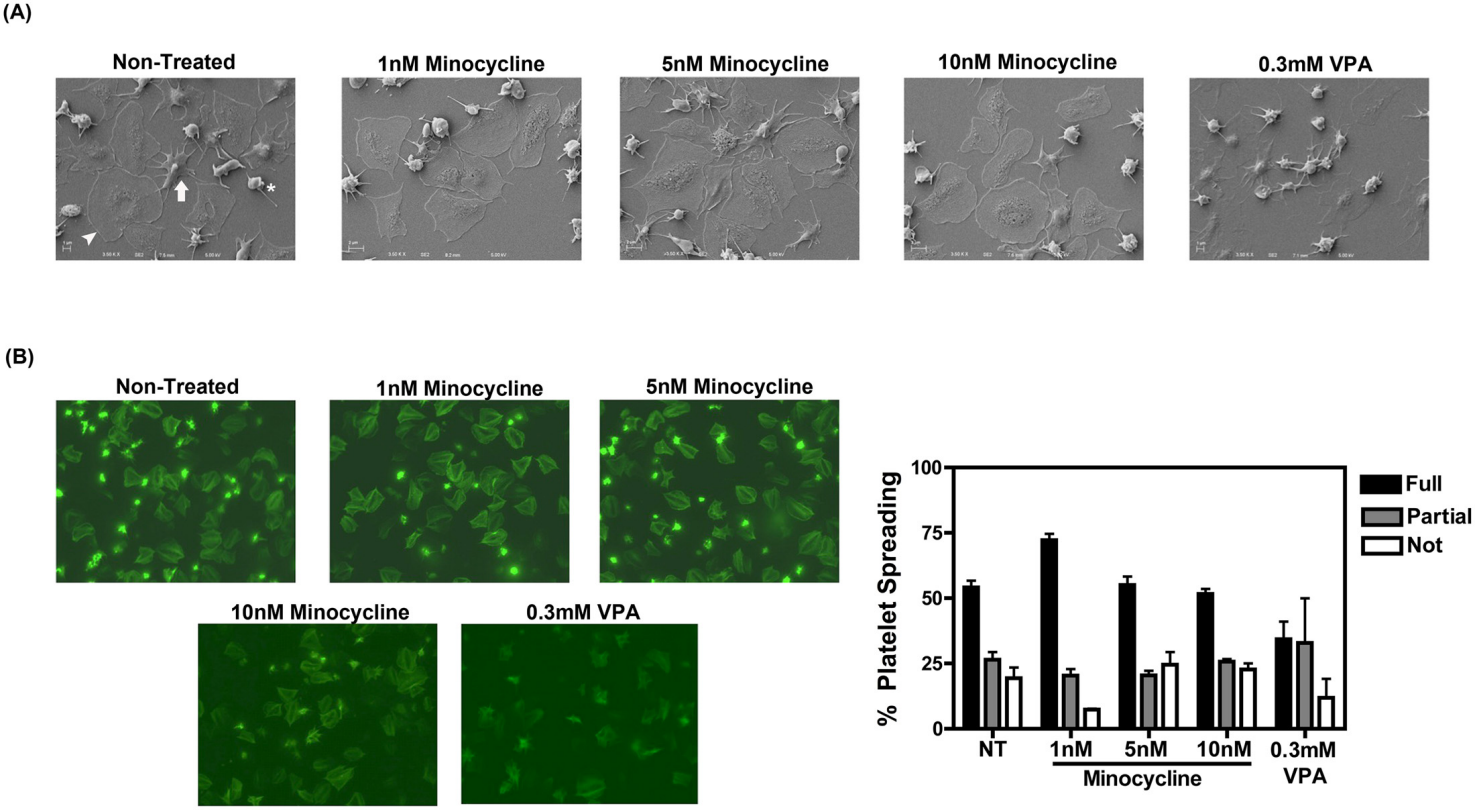
Minocycline does not affect platelet spreading. (A) Platelets were incubated with various concentrations of minocycline or 0.3mM VPA, and subsequently spread on fibrinogen-coated glass coverslips. Platelet morphology via SEM imaging is denoted as not spread (asterisk), partially spread with filopodia but not lamellopodia (arrow), or fully spread with lamellopodia (arrow head). (B) Platelets were again treated with minocycline or VPA, and spread on fibrinogen coated coverslips. Platelet spreading was assessed and quantified based on cellular morphology as described in (A). Data is representative of three separate experiments and was analyzed using a one way ANOVA statistical test. Phalloidin (green) staining of F-actin cytoskeleton was used to aid in visualization.

Another characteristic of activated platelets is their ability to adhere to and spread on extracellular constituents such as fibrinogen, collagen, and von willebrand factor, which is crucial for their role in the formation of a thrombus during wound healing [2]. Therefore, we examined whether minocycline exposure could reduce platelet spreading on fibrinogen-coated surfaces. Platelet morphology was characterized as “not spread” (those lacking visible filopodia; asterisk), “partially spread” (filopodia is present but lamellopodia is not; arrow), or “fully spread” (platelets exhibit a phenotype with only lamellopodia present; arrowhead) as indicated in Fig 3A. As shown in Fig 3B, treatment with various amounts of minocycline did not prevent platelet spreading on fibrinogen-coated surfaces despite its ability to reduce platelet granule release as shown in Fig 2. This is in contrast to platelets treated with 0.3mM VPA, which was used as a positive inhibitor of platelet spreading as previously described [45]. The quantification of spread platelets from the fluorescent images is shown adjacent to the images.

**Fig 4:**
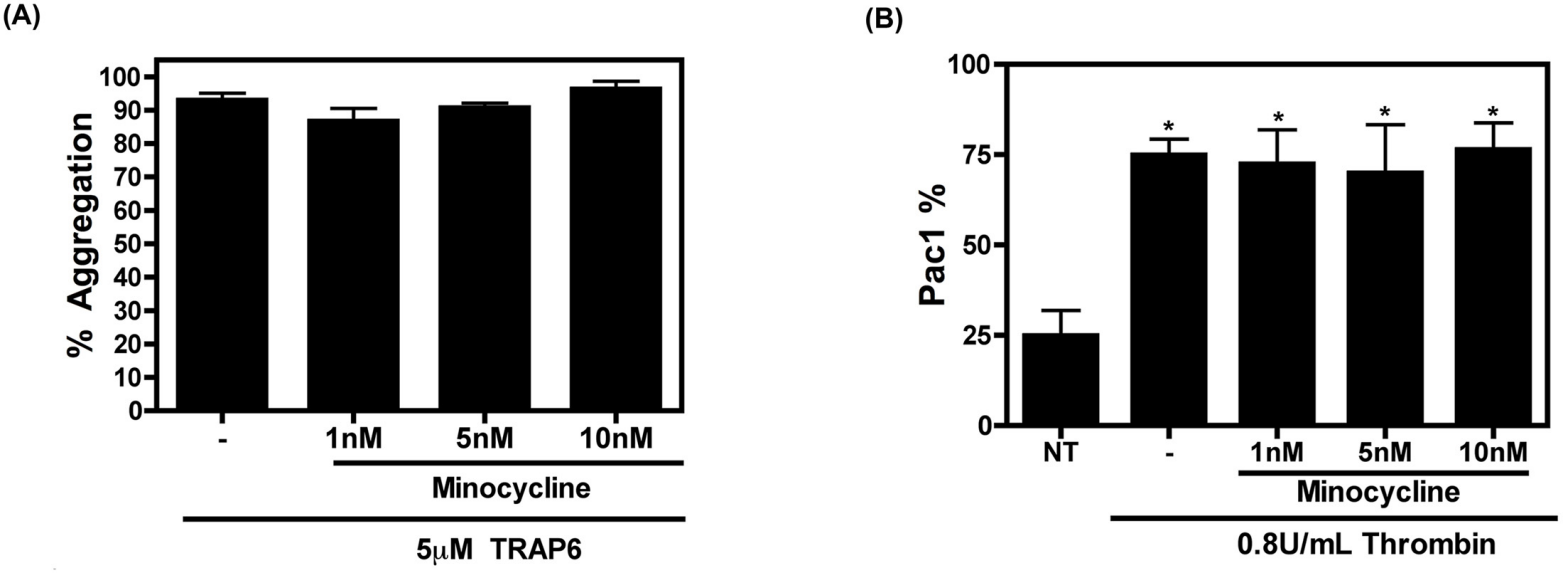
Minocycline does not affect aggregation or GPIIb/IIIa activity. (A) Platelet aggregation was measured in the presence of minocycline and TRAP 6. Percent (%) Aggregation was plotted and is representative of three separate experiments and was compared using a one way ANOVA statistical test followed by Bonferroni’s test for multiple comparisons. (B) Active GPIIb/IIIa was measured as the percentage of platelets staining positive for Pac-1 via flow cytometry. Data is representative of three separate experiments and compared using a one way ANOVA statistical test followed by Bonferroni’s test for multiple comparisons, where * denotes p<0.05.

Another function of activated platelets is the formation of homotypic aggregates that eventually develop into a thrombus [1]. We assessed the ability of platelets to aggregate after exposure to TRAP 6, a PAR 1 agonist, which acts similarly to thrombin, in the presence of minocycline. Aggregation was measured over a 6 minute time period, and was not affected by pre-treatment with minocycline (Fig 4A). Platelet aggregation and spreading are due, in part, to the activation of GPIIb/IIIa, which binds to fibrinogen and facilitates platelet-fibrinogen-platelet interaction [46]. Accordingly, we also investigated the expression of the activated form of GPIIb/IIIa after minocycline pre-treatment via flow cytometric measurement of Pac-1 staining (Pac-1 binds to the active form of GPIIb/IIIa). Exposure to thrombin resulted in an increased expression of activated GPIIb/IIIa on the surface of platelets, which was not reduced with additional minocycline treatment, thus supporting the conclusion that minocycline is unable to inhibit platelet aggregation and spreading (Fig 4B).

Collectively, these experiments demonstrate that minocycline does not inhibit all platelet functions, notably spreading and aggregation, and therefore would likely not pose a risk for bleeding associated with some antiplatelet medicines [47].

### Minocycline does not affect thrombus formation in mice

Platelet activation and subsequent release of granule contents can amplify further platelet activation, aggregation, and thrombus formation. We have shown that minocycline successfully reduces degranulation of activated platelets, but it was not rescued to levels of non-activated controls. This suggests that, although minocycline has no effect on GPIIb/IIIa modulation and aggregation, the reduction in granule release may delay thrombus formation. We therefore sought to assess the role of minocycline on thrombus formation after vascular injury.

**Fig 5:**
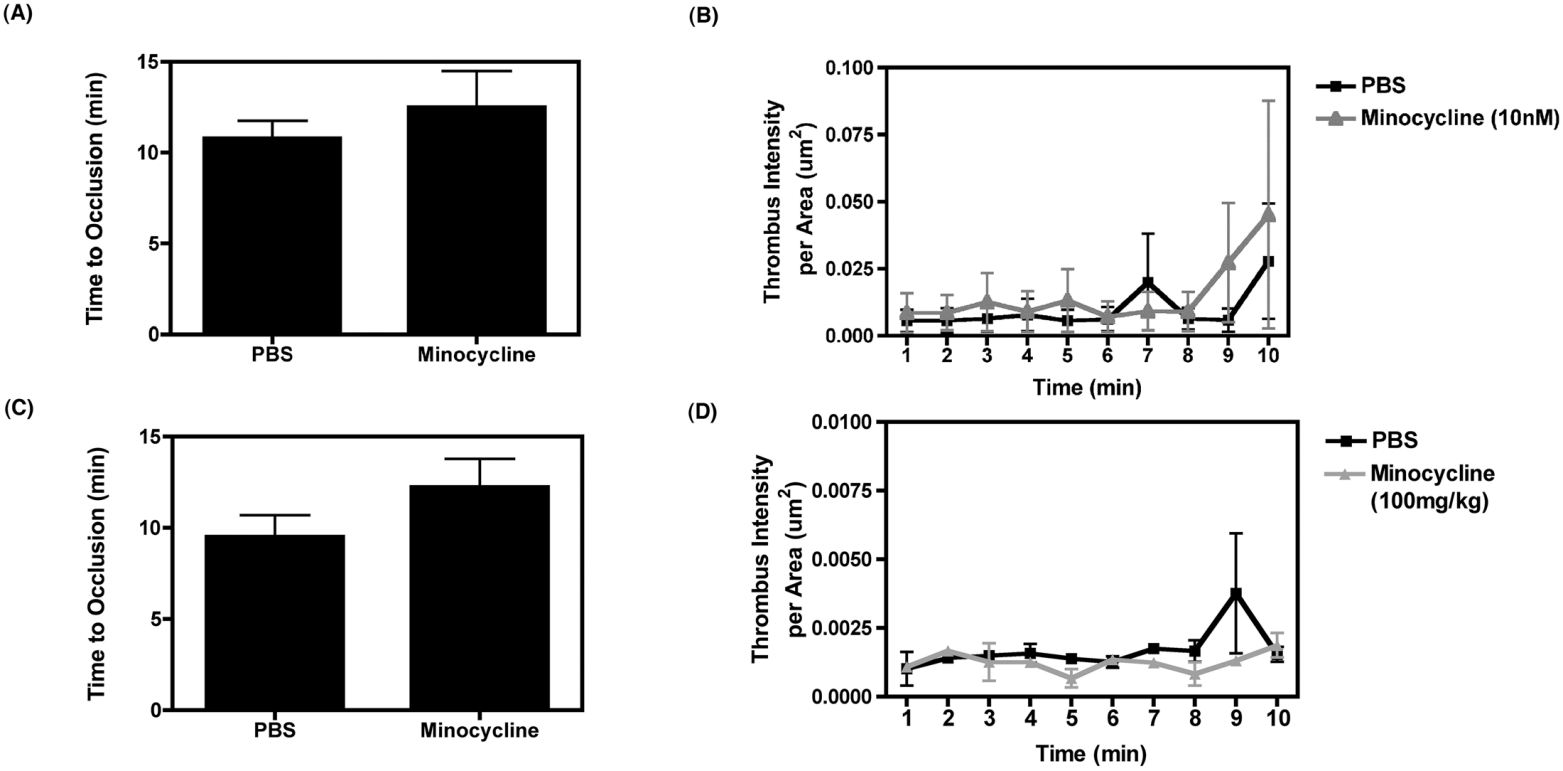
Minocycline does not affect thrombus formation. (A and B) Thrombus formation was measured in “recipient” mice (n = 6 for each group) that were injected with 10 μm calcein green–fluorescently labeled platelets that were treated with either 1X PBS or minocycline. Vascular injury was induced by application of FeCl_3_ to the mesenteric arteries for 45 s and subsequent thrombus formation was imaged and the following measurements were assessed over time: (A) the time to vessel occlusion, (B) thrombus intensity per area (μm^2^) over time. (C and D) Thrombus formation was also assessed in mice treated with 2 injections of either PBS or 100mg/kg minocycline (n = 3 for each group, injections were 12 hr apart) by measuring (C) the time to vessel occlusion and (D) thrombus intensity per area (μm^2^) over time. Data was compared using a student’s T test for (A) and (C) and a one way ANOVA test for (B) and (D) followed by Bonferroni’s test for multiple comparisons.

Platelets were isolated from control donor mice, fluorescently stained with 10 μM calcein green-AM for 30 minutes, and subsequently treated with either PBS or 10 nM minocycline for 5 minutes. The platelets were then retro-orbitally injected into “recipient” mice, where injury was induced to the mesenteric artery via topical application of FeCl_3_. Thrombus formation over time was subsequently imaged via fluorescent microscopy. As demonstrated in Fig 5A, we observed no significant difference in the time to vessel occlusion. There was also no change in thrombus intensity per vessel area between treatments (Fig 5B), suggesting that minocycline has no affect on thrombosis. We further assessed thrombus formation after systemic injection of minocycline as opposed to injection of minocycline- treated platelets; it is possible that direct, systemic minocycline treatment *in vivo* may elicit an effect on thrombosis. Thus, we performed two intraperitoneal injections of either sterile, filtered 1X PBS or 100 mg/kg minocycline approximately 12 hr apart and then measured thrombosis using the FeCl_3_ injury model. As demonstrated in Fig 5C, although there is a trend in increased occlusion time in minocycline-treated mice, the time to arterial vessel occlusion was not significantly different between PBS-treated mice and that of minocycline-treated mice. Furthermore, thrombus intensity per vessel area was also not changed amongst treatment groups (Fig 5D). This lack of difference in thrombus formation between the PBS and minocycline groups in both sets of experiments may be explained by the observation that minocycline does not affect GPIIb/IIIa activation (Fig 4), and that the minocycline-induced reduction in granule release is not to that of non-treated levels (Fig 2), thus allowing for the release of pro-aggregation signals.

Ultimately, this data suggests that minocycline selectively reduces platelet degranulation without affecting platelets’ function to spread, aggregate, and form a thrombus and would thus not pose any risk of unwarranted bleeding; minocycline may serve as a novel inhibitor of platelet-mediated inflammation (Figs 1 and 2), but may not be useful for abrogation of thrombotic or vascular occlusion disorders.

### MLK3 signaling is involved in platelet activation

p38 MAPK is activated by upstream MAPKKs, such as MAP Kinase Kinase (MKK) 3/6, which in turn can be activated by several MAPKKKs, including MLK3, ultimately following a MAPK signaling cascade [48]. Several reports have indicated that p38 MAPK is involved in platelet activation and degranulation. Despite this, no known reports have examined the role of upstream MLK3 in platelet activation. We thus wanted to first determine if MLK3, since it is upstream of p38, is involved in platelet activation.

**Fig 6:**
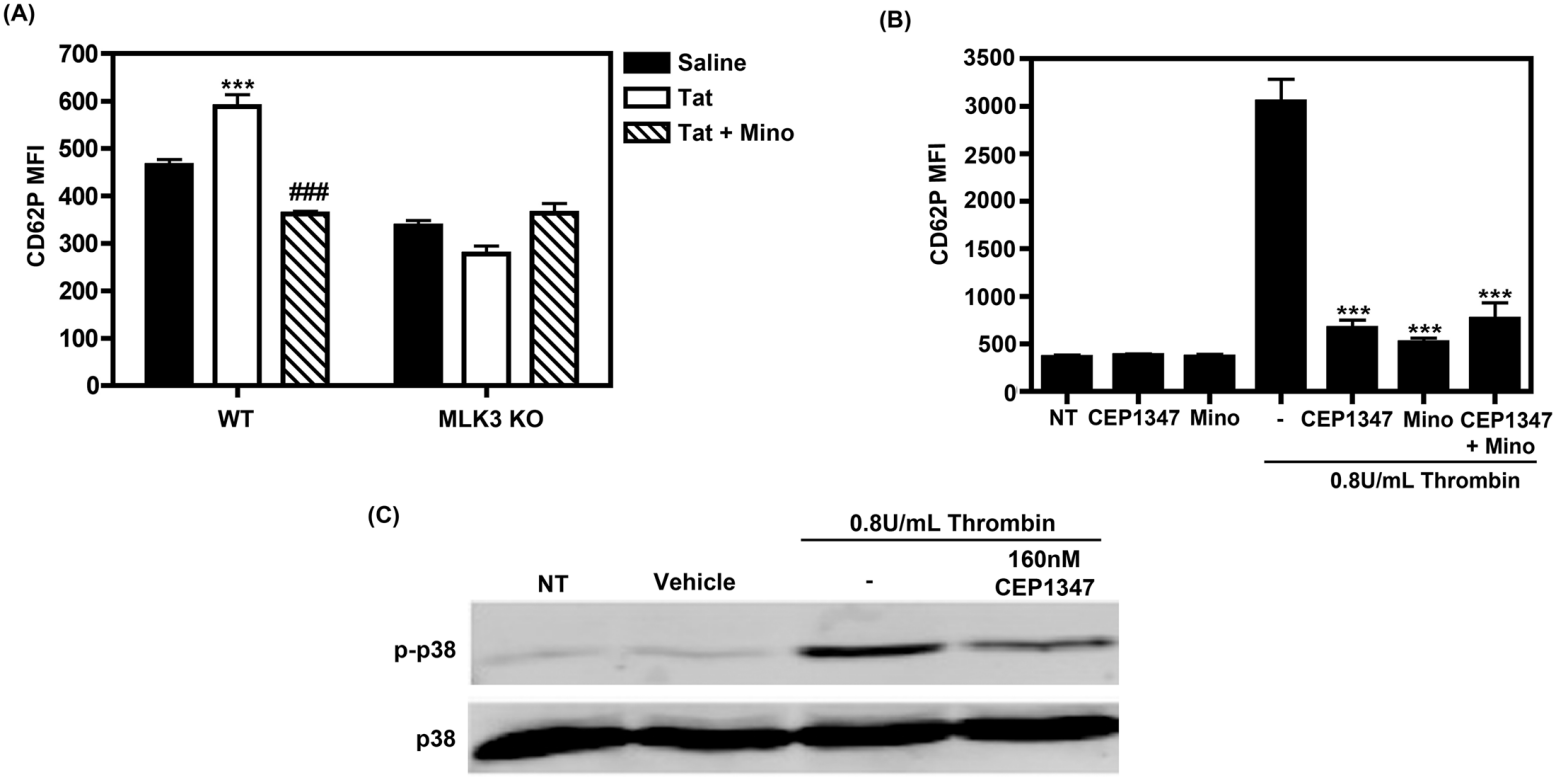
MLK3 is involved in platelet activation. (A) Platelet activation was measured by analyzing CD62P via flow cytometry from whole blood collected from WT or MLK3 KO mice after exposure to the indicated treatments (n = 4 for each group). This data set was compared using a two way ANOVA statistical test followed by Bonferroni’s test for multiple comparisons, where *** denotes p<0.0001 saline treatment compared to Tat treatment and ### denotes p<0.0001 for minocycline treatment compared to Tat treatment. (B) Isolated, washed platelets were subjected to the indicated treatments and assessed for platelet activation via CD62P MFI analysis. This data set was compared using a one way ANOVA statistical test followed by Bonferroni’s test for multiple comparisons, where *** denotes p<0.0001. Data shown is representative of three separate experiments. (C) Isolated, washed platelets were subjected to 5-minute thrombin treatment with or without increasing concentrations of MLK3 inhibitor, CEP1347, as shown. Whole cell lysates were subjected to immunoblot analysis using phosphorylated p38 MAPK or total p38 MAPK-specific antibodies. Data shown is representative of a single experiment that was repeated three times.

Wildtype (WT) or MLK3 KO mice were left untreated or injected with 100mg/kg minocycline intraperitoneally. 12 hr later mice were subsequently injected with saline or the HIV protein Tat (100ng/g body weight) retro-orbitally for 1 hr, and platelet activation was subsequently measured. We analyzed platelet activation via flow cytometric measurement of CD62P expression. As shown in Fig 6A, we observed increased CD62P expression in our WT mice treated with Tat, whereas our Tat-treated MLK3 KO mice did not show any significant change in CD62P expression, suggesting that MLK3 is involved in platelet activation. Furthermore, Tat-treated WT mice that also received minocycline therapy had a significant reduction in CD62P expression, which is lower than that of basal CD62P expression observed in our saline treated WT mice; this suggests that MLK3 activity is important for platelet activation at both basal and inducible conditions. Further, comparable levels of CD62P were observed in both the Tat + minocycline-treated WT and MLK3 KO mice, suggesting that minocycline may reduce CD62P expression via inhibition of MLK3. Indeed, overall lower CD62P expression was observed in the MLK3 KO mice as compared to the WT mice, suggesting that MLK3 activity is involved in platelet activation.

We further confirmed the importance of MLK3 in platelet activation by using a MLK3 specific inhibitor, CEP1347. Platelets were isolated from human donors and were treated with thrombin with or without 160 nM CEP1347, 5nM minocycline, or both drugs in combination. Thrombin-only treated platelets had increased CD62P expression, which was reduced with the addition of CEP1347, minocycline, and the combination of both drugs; however, no additive effect was observed in the combination therapy (Fig 6B).

Lastly, we confirmed that the MLK3-p38 signaling axis is present in platelets via immunoblot analysis. Isolated, washed human platelets were treated with thrombin with or without different concentrations of CEP1347 for 5 minutes. Whole cell lysates were subsequently subjected to immunoblot analysis for phosphorylated p38 and total p38. As indicated in Fig 6C, attenuation of MLK3 via CEP1347 led to a reduction in p38 activity.

Taken together, this data suggests that MLK3, which is an upstream MAPKKK of p38, is involved in platelet activation and that the MLK3-p38 signaling axis is active in platelets. Further, this presents MLK3 as a potential therapeutic target to reduce platelet activation and warrants further study.

### Minocycline-mediated reduction in platelet activation involves an inhibition of p38 MAPK signaling

Phosphorylation of p38 MAPK at residues Thr^180^/Tyr^182^ causes its successive activation [48, 49], resulting in phosphorylation and subsequent activation of downstream targets. Many of the anti-inflammatory properties generated via minocycline are through inhibition of the pro-inflammatory signaling molecules p38 MAPK and JNK [20, 32, 50]. Furthermore, since p38 MAPK is known to play a role in platelet activation [1, 34, 35, 40], and that minocycline’s reduction of platelet activation may target MLK3 (Fig 6A), we examined whether exposure to minocycline could also reduce p38 activity in platelets.

**Fig 7:**
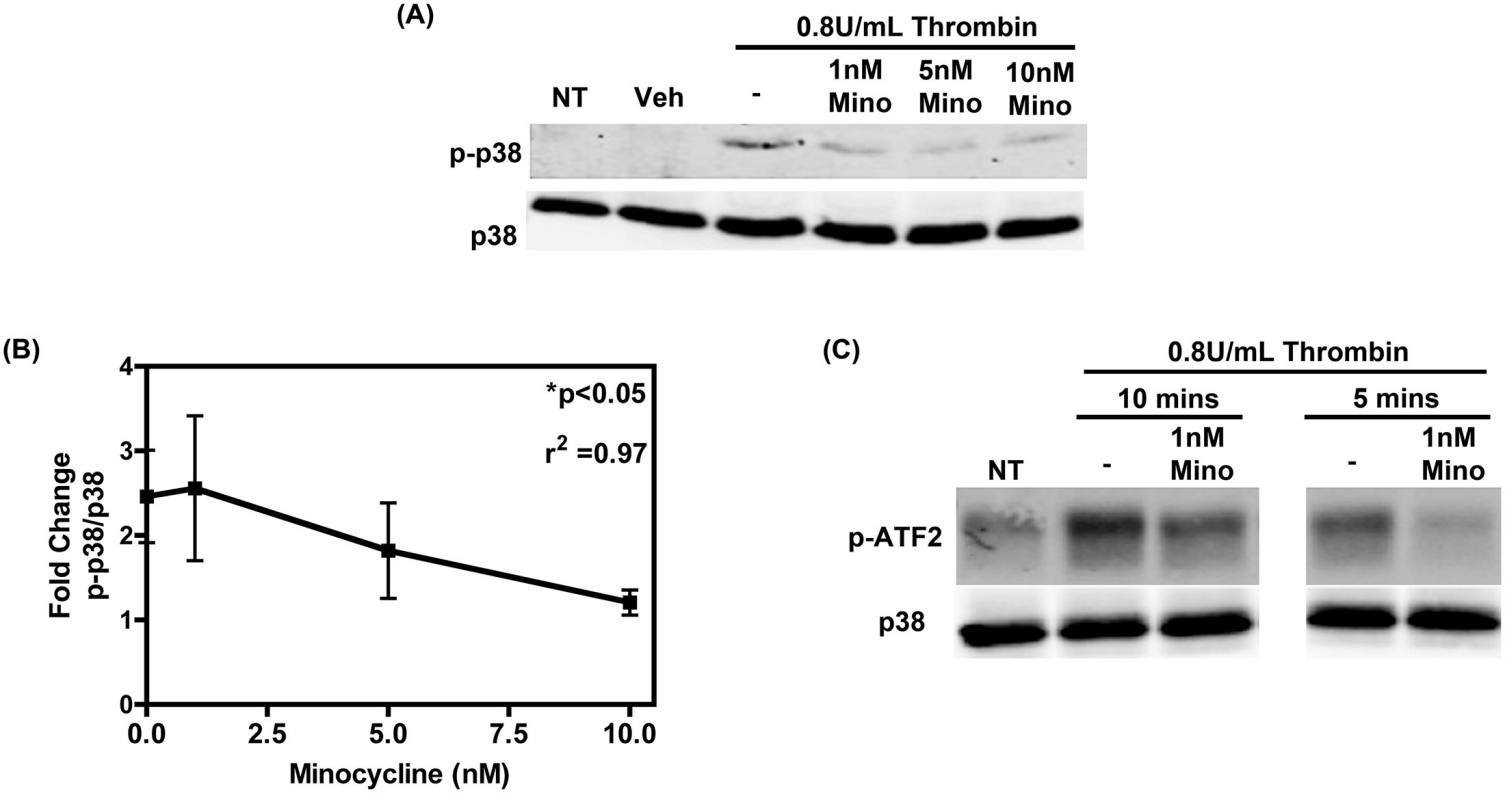
Minocycline reduces phosphorylation of MLK3 and p38 MAPK in platelets. (A) Isolated, washed human platelets were subjected to 5-minute thrombin treatment with or without increasing concentrations of minocycline pre-treatment, as indicated. Whole cell lysates were subjected to immunoblot analysis using phosphorylated p38 MAPK or total p38 MAPK-specific antibodies. Data shown is representative of a single experiment that was repeated three times. (B) Densitometric quantification of the immunoblot from (A) negatively correlates with increasing minocycline concentration. Data was compared using Pearson’s correlation statistical test with r^2^ = 0.97. (C) Platelets were treated with thrombin for 10 or 5 minutes with or without minocycline pretreatment and whole cell lysates were subsequently collected. Activity of p38 MAPK was measured via a p38 *in vitro* kinase assay using ATF-2 as a substrate, following the indicated treatments. Data shown is representative of three separate experiments.

Platelet whole cell lysates were collected, after 5-minute treatment with either thrombin alone or together with minocycline, and were subjected to immunoblot analysis for phosphorylated p38 (at residues Thr^180^/Tyr^182^) and total p38 levels. As demonstrated in Fig 7A, treatment with thrombin alone caused an increase in phosphorylation of p38. Pre-treatment with minocycline reversed this effect similar to those levels observed in non-treated platelets. Densitometric fold change values for phosphorylated p38 were calculated from the blot in Fig 7A and negatively correlated with increasing minocycline treatment (r^2^ = 0.97; Fig 7B). Since, we observed an increase in phosphorylation of p38 we therefore examined its activity using an *in vitro* kinase assay. As shown in Fig 7C, thrombin treatment for both 10 minutes and 5 minutes induced an increase in p38 activity as illustrated by an increase in phosphorylation of the p38 substrate, ATF-2. This thrombin-induced increase in p38 activity was inhibited with minocycline pre-treatment. Although it is possible that other kinases and signaling proteins may be targeted by minocycline and involved in granule release, the data presented here suggests that minocycline may exert its selective antiplatelet activity through inhibition of MLK3-p38 MAPK signaling, which may aid in the development of future selective antiplatelet therapies.

## Discussion

Despite their role in hemostasis and wound healing, abnormal platelet function and hyper-activation is known to contribute to a wide variety of inflammatory disorders, such as cardiovascular disorders, cancer, arthritis, and HIV infection, amongst others [4, 10, 13, 16].

Several antiplatelet and anticoagulant drugs are used clinically to treat various cardiovascular disorders such as thromboembolisms, ischemia/reperfusion, atherosclerosis, myocardial infarction (MI), and stroke [2]. Indeed, the cyclooxygenase inhibitor, aspirin, ADP antagonists, ticlopidine and clopidogrel, and GPIIb/IIIa inhibitors, abciximab, tirofiban, and eptifibatide, are used clinically to reduce the risks of MI, stroke, and acute coronary syndrome [51]. In the context of HIV infection, O’Brien et. al. demonstrated in a small pilot study that aspirin attenuates platelet activation, and could therefore be used as an adjunctive therapy to cART [52]. Furthermore, we have shown that VPA treatment is well tolerated and significantly reduces platelet activation in HIV-infected individuals and could thus be adjunctive to cART [45]. A drawback of these current antiplatelet agents is the potential risk of bleeding and hemostatic dysfunction due to the dampening of platelets’ ability to aggregate and form a thrombus [53]. Additionally, it has been reported that several patients develop drug resistance to aspirin and clopidogrel dual therapy [54], and therefore the development of newer, selective antiplatelet agents is warranted.

In the present study we have proposed a novel repurposing of minocycline by demonstrating its ability to specifically inhibit platelet degranulation without affecting spreading, aggregation, and thrombus formation. Indeed, after two weeks of minocycline therapy given to HIV-infected individuals on a cART regimen, sCD40L and PF4 levels (and thus platelet activation) were significantly reduced compared to baseline levels (Fig 1). Further elucidation of this phenomenon was performed via *in vitro* experiments. Our data suggests that minocycline has an effect on some aspects of platelet activation such as platelet degranulation (Fig 2), but does not have an effect on others, such as platelet spreading, homotypic aggregation, nor thrombus formation (Figs 3, 4 and 5). Several studies have suggested that p38 MAPK is involved in platelet activation and degranulation. Therefore, although novel, it is not surprising, that a MAPKKK upstream of p38, MLK3, is also involved in platelet activation and that attenuation of MLK3 affects downstream p38 activity (Fig 6). This data also suggests that MLK3 could be a novel, potential therapeutic target for minimizing platelet activity. The observed selective attenuation of platelet activation demonstrated in this study may be induced by an inhibition of MLK3-p38 MAPK signaling axis (Figs 6 and 7), although we acknowledge that it is possible for other kinases and signaling molecules to be involved. Nonetheless, the work presented here may help in the development and screening of novel antiplatelet therapies that selectively block platelet–mediated inflammation without affecting hemostasis.

This novel repurposing of minocycline as an antiplatelet agent could be useful for a variety of platelet-related disorders. Minocycline, originally synthesized for its use as an antibiotic, was found to possess anti-inflammatory properties as well, and has been studied for its therapeutic effects in rheumatoid arthritis, neurodegenerative disorders, cardiovascular disorders, and HIV infection [20]. Minocycline is lipid soluble and was found to easily penetrate the BBB into the central nervous system (CNS), hence making it an attractive drug for several CNS-related inflammatory disorders. Although it was found to have detrimental effects on patients with amyotrophic lateral sclerosis [55], minocycline treatment has shown promise in clinical trials for the treatment of multiple sclerosis (MS) and is well tolerated in MS patients [28, 29]. Furthermore, although several groups demonstrated the drug’s efficacy in reducing inflammation related to HIV infection [32, 50], it was ultimately concluded in a randomized trial study to not have an effect on cognitive improvement in patients with HIV associated neurocognitive disorder [42]. The anti-neuroinflammatory ability of minocycline may not have translated from animal models into human subjects due to the complex nature of neuroinflammation caused by HIV infection or co-infections allowing for an inflammatory response and neurotoxic environment to persist in the CNS even with minocycline treatment. Despite this apparent setback, the ability of minocycline to reduce inflammation is effective and is still being used in studies to reduce HIV-related chronic inflammation [56].

The selective inhibition of platelet function mediated by minocycline’s antagonism of p38 MAPK signaling is not surprising considering that previous reports have demonstrated that blocking p38 MAPK signaling by specific inhibitors reduces platelet granule release, yet does not affect aggregation nor spreading [34, 40, 57, 58]. These characteristics make minocycline an attractive drug to antagonize platelet activation in two ways. First, as mentioned above, a large drawback for any potential antiplatelet drug is its ability to cause excessive bleeding and hemostatic dysfunction [59]. Minocycline’s inability to abrogate spreading and aggregation of platelets, which is likely due to its incapacity to reduce activated GPIIa/IIIb (Fig 3), suggests that this drug does not pose a significant risk for bleeding disorders and the abolishment of a thrombus, which may occur via treatment with other antiplatelet agents. Secondly, the prevention of alpha and dense granule release by minocycline will result in a reduction in pro-inflammatory molecules released upon platelet activation. Admittedly, as shown in Fig 2, minocycline reduced granule release as compared to thrombin only treated platelets, but it is not rescued to that of non-treated levels. These retained levels of granule release may serve as “just enough” granule content, in addition to minocycline’s inability to block active GPIIb/IIIa, to contribute to minocycline’s overall inefficiency in blocking aggregation, spreading and thrombus formation. This selective inhibition of platelet degranulation but not aggregation suggests that, although minocycline may not be an ideal drug for the abolishment of thrombus-related hemostatic disorders such as thromboembolisms, it could be useful in reducing platelet-mediated inflammation, ultimately helping to reduce the chronic inflammation that, for example, persists in HIV infection.

Minocycline seems to be well tolerated in HIV-infected individuals [42]. Amongst other complications related to HIV, it is established that HIV-infected patients are susceptible to a dysfunction in the epithelial layer within the gut, resulting in an increase in gut associated microbes released into peripheral blood and subsequent immune cell activation [60]. With its approval as an antimicrobial drug as well as its apparent anti-inflammatory properties, minocycline may be an attractive drug for many HIV-related complications as compared to other known anti-inflammatory and antiplatelet drugs, due to its ability to combat chronic inflammation as well as bacterial infection.

Sepsis is also known to induce neutrophil recruitment and activation mediated by platelet-derived sCD40L [8], and could therefore benefit from minocycline’s antibiotic and antiplatelet effects in reducing platelet activation as well as systemic bacteremia. Additionally, cancer patients that undergo chemotherapy are at an increased risk of opportunistic infection (bacterial and viral) due to the adverse immunosuppressive effects of cancer treatment [61]. As mentioned previously, platelets aid in tumor growth [13], and therefore cancer patients may benefit from minocycline’s antibiotic and antiplatelet properties.

Minocycline’s broad-spectrum effects and high lipid solubility have supported its use as an experimental drug for *in vitro* and *in vivo* work on several inflammatory disorders including various CNS pathologies and neuropathic pain, atherosclerosis, ischemia, rheumatoid arthritis, inflammatory bowel disease, osteoporosis, and HIV [20]. Thus, minocycline, which is already approved for its use as an antimicrobial, is an attractive and novel antiplatelet agent, with its ability to prevent the release of platelet granule contents without affecting platelet spreading and aggregation. With its previously established antibiotic and anti-inflammatory properties minocycline is an appealing novel agent to selectively treat a wide variety platelet-mediated inflammatory disorders, without the risk of adverse bleeding, signifying the need for novel, selective antiplatelet therapies, and thus warrants continued study.
